# Aln2tbl: building a mitochondrial features table from a assembly alignment in fasta format

**DOI:** 10.1080/23802359.2021.1966334

**Published:** 2021-08-24

**Authors:** Joan Pons, Juan José Ensenyat, Pere Bover, Miquel Serra, Francesco Nardi

**Affiliations:** a Departament de Biodiversitat Animal i Microbiana, Institut Mediterrani d’Estudis Avançats (IMEDEA, CSIC-UIB), Esporles, Spain; b Tecnologies de la informació i la comunicació, Institut Mediterrani d’Estudis Avançats (IMEDEA, CSIC-UIB), Esporles, Spain; c Instituto Universitario de Investigación en Ciencias Ambientales (IUCA), Grupo Aragosaurus - Departamento de Ciencias de la Tierra, Facultad de Ciencias, Universidad de Zaragoza, Zaragoza, Spain; d Department of Life Sciences, University of Siena, Siena, Italy

**Keywords:** Mitochondrial genome, gene annotation, feature table, Python, GenBank submission

## Abstract

The sequencing, annotation and analysis of complete mitochondrial genomes is an important research tool in phylogeny and evolution. Starting with the primary sequence, genes/features are generally annotated automatically to obtain preliminary annotations in the form of a feature table. Further manual curation in a graphic alignment editor is nevertheless necessary to revise annotations. As such, the automatically generated feature table is invalidated and has to be modified manually before submission to data banks. We developed aln2tbl.py, a python script that recreates a feature table from a manually refined alignment of genes mapped on the mitochondrial genome in fasta format. The feature table is populated with notes and annotations specific to mitochondrial genomes. The table can be used to create a sqn file to be submitted directly to data banks. In summary, our scripts fills one gap in the available toolbox and, combined with other software, allows the automation of the entire process, from primary sequence to annotated genome submission, even if a manual curation step is conducted in a visual sequence editor.

## 1. Introduction

The collection of complete mitochondrial genome sequences from a genome project at low coverage is now straightforward due to advances in high-throughput sequencing. Several bioinformatic pipelines were developed to automatically annotate the 37 genes generally encoded in animal mitogenomes, with MITOS (Bernt et al. 2013) and MITOS2 (Donath et al. 2019) being a popular option. This pipeline puts together BLAST searches based on sequence identity and hidden Markov models (HMM) to annotate protein and ribosomal coding genes, and covariance models based on cloverleaf-like structures and other relaxed models to annotate tRNA coding genes. Other programs were designed to find mitochondrial genes with intron sequences (Mfannot; Lang et al. 2007), to work on specific taxa such as fish (MitoAnnotator; Iwasaki et al. 2013) or seed plants (MITOFY; Alverson et al. 2010), to annotate both organelle genomes (GeSeq; Tillich et al. 2017; and GetOrganelle; Jin et al. 2020) and even to perform the full pipeline from reads assembly, gene annotation and mitogenome visualization in a single tool (MitoZ; Yang et al. 2019). These annotation programs generally produce a feature table that could be used to submit mitogenome annotations to data banks directly using online (e.g. Webin and Bankit) and offline tools (e.g. Sequin and tbl2asn). The feature table used by DDBJ/ENA/GenBank (Definition Version 10.9 November 2019) includes all gene annotations as a five-column, tab-delimited table of feature locations and qualifiers.

Nevertheless, automatic annotations generally require some sort of manual curation in a visual alignment editor, as the comparison of gene sequences w.ecies may reveal inconsistencies, particularly at the beginnings and ends of CDS and ribosomal genes, less frequently in tRNA genes. Manual curation of an alignment of individual genes mapped on complete mitochondrial sequences in fasta format is a fairly straightforward process that can be appropriately performed in visual alignment editors such as Aliview (Larsson 2014) and Seaview (Gouy et al. 2010). Editing, nevertheless, invalidates the automatically produced feature table and introduces the need to manually update the table based on modifications manually introduced in the alignment. Minor corrections of locations and qualifiers in the feature table are easily accomplished manually, but continuous refinement, update, addition and deletion of gene features is a lengthy process prone to errors. A new workaround was recently proposed in the context of mitoconstrictor (Lubośny et al. 2020) to update feature tables from sequence files in gbk format. We propose a new python3 script aln2tbl.py that, starting from a modified alignment in fasta format, such as those produced in Aliview and Seaview, recreates the corresponding feature table as a standard five-column and tab-delimited table compatible with downstream applications. The feature table is further populated with information specific to the mitochondrial genome. Notably, this feature table file can be used as input in tbl2asn, along with the complete nucleotide sequence of mitogenome and the submitter information, to automatically create a sqn file to be directly submitted to data banks. The new script fills a gap in the available pipeline and allows the full automation of the procedure from primary sequence to genome submission (apart from the actual step of manual visualization/editing of annotations). This should foster manual curation of newly sequenced genomes, something that is emerging as a priority in the field of mitochondrial genomics.

## 2. Materials and methods

The aln2tbl.py script is written in python v3.8.3 and is freely available, including documentation and examples, from GitHub (https://github.com/IMEDEA/mitogenomics). An older python v2 version is also available in GitHub but will not be maintained further. The script requires two python modules (biopython and argparse) as dependencies. As long as the python interpreter and dependencies are installed, the script is platform independent. See the Results section and GitHub page for additional details on implementation and usage.

The script aln2tbl.py was tested on 35 mitogenomes from different taxonomic groups (see Supplementary Table 1) downloaded from GenBank. Individual genes were extracted based on the original GenBank annotations to simulate a MITOS output. A mitogenome contig was constructed in Bowtie2 (Langmead and Salzberg 2012) to be visualized in Aliview. The new script aln2tbl.py was used to recreate the corresponding feature table. This, alongside the primary sequence and ancillary information, was used as input for tbl2asn (v25.8) to create a sqn file ready for submission and the corresponding GenBank file. This latter was compared with the original GenBank file deposited in NCBI to assess script accuracy.

## 3. Results

### 3.1. Implementation and usage

The aln2tbl.py script takes as input (a) a fasta file including, as separate sequences, the entire genome and each subsequence to be annotated, generally the 37 genes and a control region; (b) a text file listing genes that are encoded on the sequenced strand; (c) an indication of the genetic code to be used. The script can be run using the following command:

aln2tbl.py -f assembly_file.fas -g forward_genes_file.txt -c number_genetic_code > feature_table_file.tbl

This assumes python3 is the default interpreter in the environment /usr/bin/env python3. If this is not the case, or multiple python installations are available, the full path to python3 interpreter can be added to the command line (e.g. /usr/bin/python3 aln2tbl.py).

The fasta alignment (contig or assembly) file (-f/–fasta) must include the complete nucleotide sequence of the mitogenome as well as the sequence of each mapped gene aligned below, with a single line per gene sequence. Gene names must comply with the names proposed by Boore and Brown (2000) as in most recent GenBank annotations. Names for the protein coding genes are as follows: atp6, atp8, cob, cox1, cox2, cox3, nad1, nad2, nad3, nad4, nad4L, nad5 and nad6. Accepted names for ribosomal genes are rrnL (or 16S) and rrnS (or 12S) for the large and small ribosomal subunit, respectively. tRNAs are indicated using the single letter corresponding to the encoded tRNA (e.g. M for methionine). The two tRNA genes that are generally present for Leucine and Serine must be differentiated by post-pending the correct number to the gene name: L1 for *tRNA^Leu(CUN)^*, L2 for *tRNA^Leu(UUR)^*, S1 for *tRNA^Ser(AGN)^* and S2 for *tRNA^Ser(UCN)^*). The control region, if included, must be named 'control_region' or 'CR'. If two or more control regions are present, they can be differentiated and named as 'CR1' and 'CR2'. Control regions, like PCGs, rRNAs and tRNAs, should be identified based on strong evidences prior to annotation such as position, base composition, and presence of specific conserved elements. Hence, we suggest not to annotate any long non-coding region as control region. Any additional annotation, if present, will be annotated as miscellaneous feature. In order to produce a well organized and easily readable feature table, gene sequences should be sorted by the position of the first nucleotide. If not, they will nevertheless be reordered automatically by tbl2asn at a later stage (see below).

The initial fasta alignment of automatically annotated gene sequences against the complete mitogenome can be obtained in several ways starting from the results of an automatic annotation (e.g. MITOS). One flexible option, using free software that can be called using bash scripting, is to build the contig using Bowtie2, sort the bam file by coordinate in picard tools (http://broadinstitute.github.io/picard), and finally convert the bam file to fasta with the python3 script sam2fasta.py (https://sourceforge.net/projects/sam2fasta/files/). Another option, provided that gene and genome sequences are position ordered, identical and ungapped (e.g. in the MITOS output), is to use the python v3 script mitos2aln.py, provided as companion to aln2tbl.phy. This script produces a fasta alignment of each gene nucleotide sequence relative to complete mitogenome sequence and parses MITOS gene nomenclature of fasta headers with four semicolon separated fields to single names compatible with aln2tbl.py naming (see below for duplicated genes). This fasta contig with automatic gene annotations obtained in MITOS can now be the easily refined manually in a visual alignment editor such as SeaView or Aliview prior to further processing in aln2tbl.py.

The second argument (-g/–genes) takes a plain text file with the list of genes encoded on the sequenced strand (i.e. in forward orientation in the sequence that is being submitted), identified by name, separated by commas and without spaces. This information allows the script to correctly reverse/complement the nucleotide sequence of genes encoded on the opposite strand prior to annotation. The control region should be also included in the gene file (–genes) if it has to be annotated in forward orientation.

The last argument (-c/–code) parses the number of the appropriated mitochondrial genetic code (e.g. 2 for vertebrate mitochondrial code, 5 for invertebrate mitochondrial code; see https://www.ncbi.nlm.nih.gov/Taxonomy/Utils/wprintgc.cgi for a complete list). The output should be saved as a text file with tbl extension to be readily identified as a feature table.

The aln2tbl.py script is able to annotate non-canonical start and truncated stop codons in CDS genes correctly as translation exceptions. An appropriate description is added as a qualifier note following standard practices. Both truncated stop codons (TA, T) are correctly processed. Nevertheless the use of truncated TA stop codons is discouraged as this is not recognized by some downstream applications (e.g. tbl2asn). A truncated T codon can be used as a substitute. The script also detects CDS ending with a non-canonical stop codon and prints an error in the output feature table (“ERROR IN STOP CODON”) so input alignment can be corrected and the script run again. The aln2tbl.py script can not handle partial or split genes, though this is an uncommon circumstance in mitogenomes. If present, the feature table must be manually corrected by adding the symbols < and > for partial 5′ and 3′ ends, respectively. The presence of duplicated tRNA genes, which is also uncommon, cannot be handled as it results in a duplicated gene name, and must be manually annotated in the feature table.

Once the final feature table is created with aln2tbl.py, the program tbl2asn (https://www.ncbi.nlm.nih.gov/genbank/tbl2asn2/) can be used to generate a sqn file to be submitted to data banks. The program tbl2asn takes as input: (a) the complete genome sequence in fasta format (-i argument, with extension fas), which header should correspond to the genome sequence name in the feature table, and may optionally include source modifiers in square brackets (e.g. topology and specimen-voucher; see https://www.ncbi.nlm.nih.gov/Sequin/modifiers.html for a complete list). Alternatively, source modifiers can be added using the -j command; (b) the feature table (-f command, with extension tbl) as recreated using aln2tbl.py; (c) submitter and manuscript information (-t argument, with extension sbt) that can be prepared using the NCBI web service https://submit.ncbi.nlm.nih.gov/genbank/template/submission/. The program tbl2asn may also include the sub-command -T to remotely check and include full taxonomy of the species. Additional details on tbl2asn can be found at its home page on NCBI: https://www.ncbi.nlm.nih.gov/genbank/tbl2asn2/. The script can be run using the following command:

tbl2asn−i mitogenome.fas−f featuretable_file.tbl−t submission_template.sbt−a s−V bv−T−j"[mgcode=5][location=mitochondrion][organism=Hyalella solida]"

**Figure 1.**
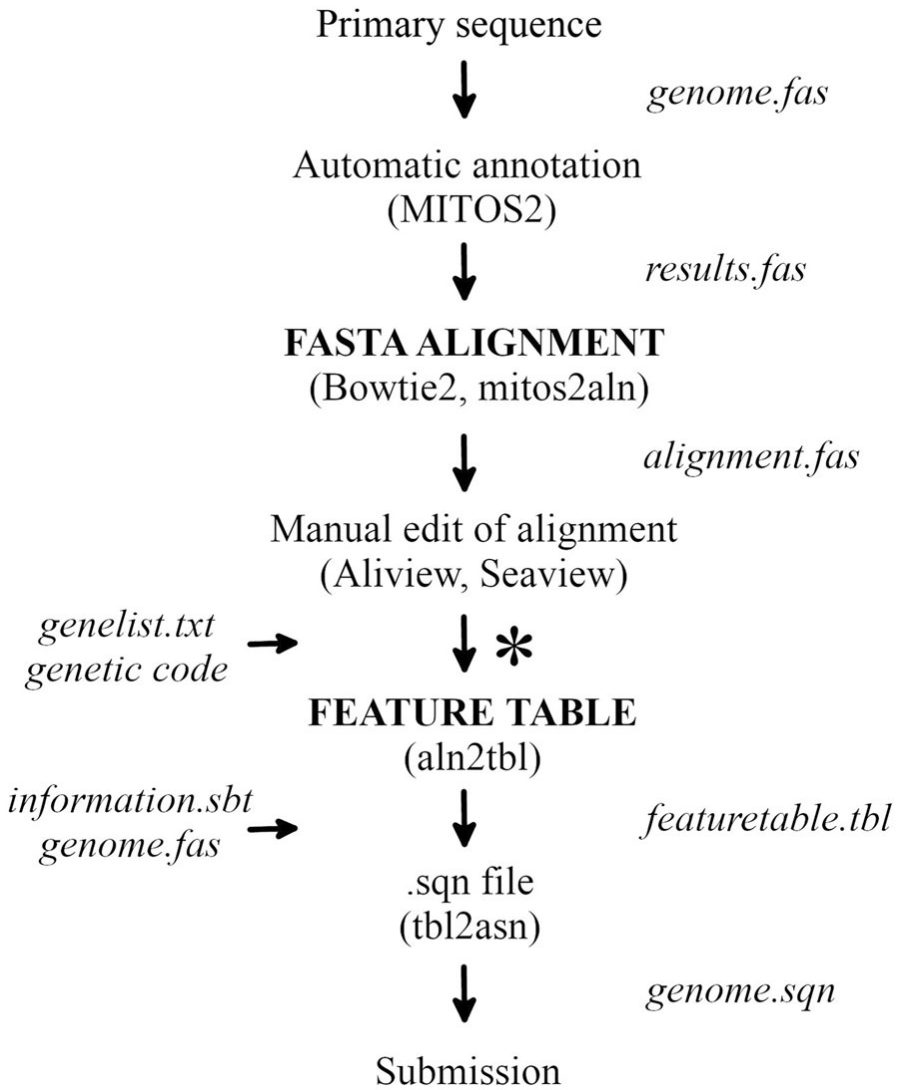
Procedure from primary mitochondrial sequence to submission to GenBank. *Indicates the position of the aln2tbl script.

This will produce four files. The file mitogenome.sqn is the main output file and can be used to directly submit the annotated genome sequence to GenBank. The file mitogenome.gbf corresponds to the GenBank record in human readable format, as it will be visible after processing and publication, while mitogenome.val includes warnings or errors. The file errorsummary.val also summarize errors. The pipeline described here is summarized in Figure 1 and a full example, scripts and pipeline are deposited in GitHub.

### 3.2. Testing

Comparison of the 35 genomes annotations produced using aln2tbl.py with the original GenBank annotation of the same genomes (Supplementary Table 1, Supplementary Material) produced no inconsistency overall, indicating that the script is fully functional and capable of addressing idiosyncrasies of individual genome annotations from different lineages. The programs aln2tbl.py and tbl2asn can not handle duplicated genes with identical name so this issue must be solved manually.

## 4. Discussion

The manual curation of automatic annotations is a necessary step in the process of genome analysis prior to submission to a databank. Manual editing in a visual sequence editor nevertheless invalidates the automatically produced feature table. Our script aln2tbl.py can easily recreate the feature table based on the modified contig and allows further processing in tbl2aln to produce a sqn file that can be directly submitted to databanks. Besides filling a gap in the current pipeline, allowing the automation of the entire process using scripting, aln2tbl.py is specifically designed with mitochondrial genomes in mind. It can handle non canonical start and stop codons (e.g. 'transl_except (pos:11922,aa:TERM)'), it introduces annotations specific to the mitochondrial genome following current good practices in the field (e.g. 'TAA stop codon is completed by the addition of 3′ A residues to the mRNA') and transforms abbreviated gene names into full names in the product field (e.g. 'product NADH dehydrogenase subunit 3′). We hope that, by facilitating the entire process, more authors will introduce a manual curation step of annotations in their production pipelines, something that is frequently advocated but not always accomplished (Prada and Boore 2019; Cucini et al. 2021).

## Supplementary Material

Supplemental Material

## Data Availability

The Python scripts aln2tbl.py, alongside its companion mitos2aln.py, documentation and examples, is freely available on GitHub (https://github.com/IMEDEA/mitogenomics) under the license GNU GPLv3.
