# Supplementary material for: Aln2tbl: building a mitochondrial features table from a assembly alignment in fasta format
Source: Mitochondrial DNA B Resour. 2021 Aug 24;6(9):2732–5. doi: 10.1080/23802359.2021.1966334 (PMC8386716; doi:10.1080/23802359.2021.1966334)
Supplement: Supplemental Material [file TMDN_A_1966334_SM8066.docx]

Supplementary Table 1: List of genomes used to test the aln2tbl.py script.

| Accession | Species | Lineage |
| --- | --- | --- |
| NC_007789 | *Acanthaster brevispinus* | Echinodermata |
| NC_024726 | *Abrornis inornata* | Vertebrata |
| NC_008452 | *Acanthocardia tuberculata* | Mollusca |
| NC_039685 | *Laqueus japonicus* | Brachiopoda |
| NC_010197 | *Bugula neritina* | Bryozoa |
| NC_026997 | *Allonautilus scrobiculatus* | Mollusca |
| NC_013811 | *Decipisagitta decipiens* | Chaetognatha |
| NC_011016 | *Acanella arbuscula* | Cnidaria |
| NC_029168 | *Acasta sulcata* | Crustacea |
| NC_045305 | *Beroe cucumis* | Ctenophora |
| NC_010431 | *Loxocorone allax* | Entoprocta |
| NC_011943 | *Abalistes stellaris* | Vertebrata |
| NC_024601 | *Achatina fulica* | Mollusca |
| NC_026985 | *Lepidodermella squamata* | Gastrotricha |
| NC_038132 | *Aacanthocnema dobsoni* | Insecta |
| NC_031873 | *Echinoderes svetlanae* | Kinorhyncha |
| NC_005212 | *Acinonyx jubatus* | Vertebrata |
| NC_005828 | *Caenolestes fuliginosus* | Vertebrata |
| NC_021932 | *Abacion magnum* | Diplopoda |
| NC_016197 | *Acanthocheilonema viteae* | Nematoda |
| NC_044095 | *Gordionus alpestris* | Nematomorpha |
| NC_023929 | *Amphiporus formidabilis* | Nemertea |
| NC_025292 | *Amynthas aspergillus* | Annelida |
| NC_009082 | *Epiperipatus biolleyi* | Onychophora |
| NC_041549 | *Polyplacotoma mediterranea* | Placozoa |
| NC_030339 | *Aglaiogyrodactylus forficulatus* | Platyhelminthes |
| NC_046790 | *Aphrodita australis* | Annelida |
| NC_010213 | *Agelas schmidti* | Porifera |
| NC_020030 | *Halicryptus spinulosus* | Priapulida |
| NC_010484 | *Brachionus plicatilis* | Rotifera |
| NC_005958 | *Abronia graminea* | Vertebrata |
| NC_014848 | *Hypsibius dujardini* | Tardigrada |
| NC_013584 | *Aplidium conicum* | Tunicata |
| NC_006688 | *Alytes obstetricans pertinax* | Vertebrata |
| NC_033971 | *Agelena silvatica* | Chelicerata |
